# Construction and application of a nomogram model for early stage central lymph node metastasis in papillary thyroid cancer combined with Hashimoto's thyroiditis

**DOI:** 10.1590/1414-431X2025e14881

**Published:** 2026-01-09

**Authors:** Ying Huang, Fanyu Zeng, Jinshuang Song, Yong Du

**Affiliations:** 1 Department of Breast and Thyroid Surgery, Affiliated Hospital of Guilin Medical University, Guilin, Guangxi, China

**Keywords:** Papillary thyroid cancer, Hashimoto's thyroiditis, Nomogram, Lymph node metastasis, Central cervical region

## Abstract

This study aimed to explore the construction and application of a nomogram model for early-stage cervical central lymph node metastasis (CLNM) in papillary thyroid cancer (PTC) combined with Hashimoto's thyroiditis (HT). The study included 360 patients with pathologically diagnosed PTC. Of these, 100 patients had PTC with concurrent HT. Univariate and multivariate analyses were conducted to identify risk factors for CLNM in patients with PTC and HT. A nomogram was designed to guide clinical decision-making. Age, gender, thyroid peroxidase antibody status, tumor diameter, tumor location, multifocal lesions, capsular invasion, calcification, aspect ratio ≥1, irregular morphology, and tumor diameter ≥1 cm strongly correlated with CLNM in patients with PTC combined with HT. Notably, capsular invasion, aspect ratio ≥1, calcification, and irregular morphology were risk factors for CLNM in patients with PTC combined with HT. A nomogram was constructed to visualize and graphically calculate the probability of CLNM development in these patients. Our findings indicated that the independent risk factors for CLNM development in patients with PTC combined with HT are capsular invasion, aspect ratio ≥1, calcification, and irregular morphology. The nomogram model developed in this study has great potential for clinical applications.

## Introduction

Papillary thyroid cancer (PTC) is the most prevalent malignant form of thyroid cancers, and its incidence is rapidly increasing (1). PTC is frequently found in patients with Hashimoto's thyroiditis (HT) (2). HT is a common autoimmune endocrine disorder responsible for most hypothyroidism cases in regions with adequate iodine intake (3). About 20-30% of HT cases are thought to be caused by a combination of environmental factors and genetic susceptibility, which results in the loss of immunological tolerance, thereby leading to an autoimmune attack on the thyroid tissue (4). HT may be considered a protective factor for PTC, leading to less invasive disease and a lower recurrence rate (5). The incidence of cervical central lymph node metastasis (CLNM) is 40-90% and is a primary risk factor for elevated recurrence (6). Moreover, patients with PTC and HT experience more lymph node dissection owing to enlarged lymphadenopathy, which may contribute to more postoperative complications (7). Therefore, distinguishing between proliferative and malignant lymph nodes before surgery is a critical step in guiding a precise surgical protocol for CLNM management.

Since its discovery by Dailey et al. (8) in 1955, the relationship between PTC and HT has been a controversial topic. Both HT and PTC exhibit some common dysregulated non-immune-linked genes involved in oxidative stress, reactive oxygen species production, cell apoptosis, and DNA damage and repair (9). At diagnosis, patients with PTC and HT usually have more limited disease and a lower frequency of extrathyroidal invasion, distant metastases, and nodal metastases than those without HT (10). Similarly, another study revealed that patients with PTC and HT had less aggressive disease and distant metastases. Additionally, patients with PTC and HT have more favorable outcomes due to less persistent disease and a higher cure rate than those with PTC but not HT (11). In contrast, Babli et al. (12) found that multifocal or bilateral lesions together with a high rate of lymph node metastasis tended to occur in patients with PTC and HT. Although HT enhances the risk of PTC, patients with PTC and HT have a less aggressive clinical presentation and decreased recurrence rate (13). Considering these, a nomogram model of comprehensive preoperative risk factors might be a novel and effective tool for identifying differential surgical strategies. Hence, this study aimed to develop a nomogram model to provide a quantitative probability of cervical CLNM in patients with PTC and HT.

## Material and Methods

### Ethics statement

This study was approved by the Ethics Committee of the Affiliated Hospital of Guilin Medical University. All participating patients and their families provided written informed consent.

### Subjects

This retrospective study included 360 consecutive patients who underwent surgical treatment and were pathologically diagnosed with PTC at the Affiliated Hospital of Guilin Medical University between July 2020 and June 2022. The inclusion criteria were as follows: 1) PTC was first operated on in this hospital and diagnosed by pathology (the pathological results of all tissues were confirmed by two expert pathologists); 2) the scope of surgical resection for PTC was at least thyroidectomy of the affected side + central lymph node dissection of the affected side; and 3) the clinicopathological data were complete and without defects. The exclusion criteria were: 1) combined or follicular thyroid cancer; 2) combined or medullary thyroid cancer; 3) combined or undifferentiated thyroid cancer; 4) second or multiple thyroid surgeries; 5) thyroidectomy without central lymph node dissection; and 6) incomplete clinicopathological data.

According to pathology results, the 360 patients with PTC were divided into: 1) PTC and HT group (HTPTC group, n=100) and 2) PTC without HT group (PTC group, n=260). Additionally, patients with HT were further categorized into subgroups according to CLNM status (the CLNM and non-CLNM groups).

### Preoperative ultrasonography

A color Doppler ultrasound diagnostic instrument (model: SIEMENS S2000 color Doppler ultrasound imaging system; USA) equipped with a high-frequency linear array probe in the frequency range of 4-9 MHZ was used. During the scan, the patient laid supine on a bed, fully exposing the anterior cervical region. The thyroid-scanning mode was selected. An ultrasound coupling gel was applied to the patient's thyroid, and a high-frequency probe was placed directly on the skin for thyroid ultrasound examination. First, a two-dimensional ultrasound was used to comprehensively scan the entire thyroid gland in multiple planes. The frequency, gain, and time-gain compensation curves were adjusted according to each patient's condition to obtain the best image quality. The depth and focus were adjusted according to the location of the nodules. Continuous scanning of longitudinal, transverse, and oblique sections of the thyroid was performed to obtain conventional ultrasound images. Data were stored and analyzed by two ultrasound physicians with more than 10 years of experience. In cases of disagreement, a senior physician participated in discussions and decision-making. Ultrasound features included: tumor diameter (the maximum diameter of the tumor), multifocality (≥2 nodules were considered multifocal), aspect ratio (<1, ≥1), longitudinal location of the tumor (upper, middle, and lower thyroid), calcification (calcified or not), capsular invasion (present/absent), and morphology (regular or not). All thyroid ultrasound examinations were performed at the ultrasound department of the hospital.

### Detection of antibody levels

The relative levels of serum thyroglobulin antibody (TgAb) and thyroid peroxidase antibody (TPOAb) were retrospectively collected from the results of the hospital testing system using a Roche Electrochemiluminescence Automatic Immunoassay System (model Cobas E601, Germany). About 3-5 mL of venous blood was acquired on an empty stomach before the operation, placed in non-anticoagulant tubes, allowed to stand for 30 min at ambient temperature, and then centrifuged at 1845*g* for 10 min at 4°C to collect the serum. Subsequently, electrochemiluminescence was used to test the levels of serum TgAb and TPOAb using a kit purchased from Wuhan Ediagnosis Biological Co. (China). The normal range for TgAb was <115 IU/mL and the normal range for TPOAb was <34 IU/mL.

### Statistical analysis

SPSS 20.0 (IBM, USA) statistical software was applied to analyze the data. Categorical data are reported as the number of cases and were compared using the chi-squared test. Measurement data are reported as means±SD and were compared by the *t*-test. P<0.05 indicated a significant difference. Univariate and multivariate logistic regression analyses were performed to determine independent risk factors for the development of CLNM in PTC and HT. A nomogram for predicting CLNM was established based on the results of multivariate logistic regression analysis.

## Results

### Clinicopathological characteristics of the HTPTC and PTC groups

The HTPTC group had a lower proportion of females, an elevated tumor aspect ratio, a reduced CLNM rate, a smaller tumor diameter, a lower probability of capsular invasion (with a greater tendency to be confined within the capsule), and higher serum TgAb and TPOAb levels compared with the PTC group (all P<0.05; Table 1).

**Table 1:** Comparison of clinicopathological characteristics of the pure PTC group and the HTPTC group.

Variable	Pure PTC group (n=260)	HTPTC group (n=100)	P
Age (years)	46.38±10.49	47.54±10.61	0.350
Gender			<0.001
Male	37 (14.23)	55 (55.00)	
Female	223 (85.77)	45 (45.00)	
Aspect ratio			0.014
<1	194 (74.62)	61 (61.00)	
≥1	66 (25.38)	39 (39.00)	
CLNM			0.003
Yes	78 (30.00)	15 (15.00)	
No	182 (70.00)	85 (85.00)	
Tumor diameter (cm)			<0.001
<1	113 (43.46)	66 (66.00)	
≥1	147 (56.54)	34 (34.00)	
Capsular invasion			0.019
Yes	82 (31.54)	19 (19.00)	
No	178 (68.46)	81 (81.00)	
Tumor extension			0.038
Confined into the capsule	178 (68.46)	81 (81.00)	
Extension to the capsule	59 (22.69)	16 (16.00)	
Extension to adjacent structures	23 (8.85)	3 (3.00)	
TgAb (IU/mL)	59.27±10.12	541.18±166.22	<0.001
TPOAb (IU/mL)	20.87±2.08	346.49±30.83	<0.001

Data are reported as means and SD or number (%). Student's *t*-test and chi-squared test. PTC: papillary thyroid cancer; HTPTC: Hashimoto's thyroiditis + papillary thyroid cancer

### Clinical data of the CLNM group and the non-CLNM group among patients with PTC and HT

Among the 100 patients with PTC and HT, 15 had CLNM and 85 did not. The CLNM and non-CLNM groups differed significantly in age, gender, and TPOAb status (all P<0.05; Table 2).

**Table 2:** Comparison of clinical data and preoperative ultrasound features of the central lymph node metastasis (CLNM) group and the non-CLNM group among papillary thyroid cancer (PTC) patients combined with Hashimoto's thyroiditis (HT).

	Non-CLNM group (n=85)	CLNM group (n=15)	P
Age (years)	48.50±10.34	42.11±10.83	0.031
Gender			0.017
Male	51 (60.00)	4 (26.67)	
Female	34 (40.00)	11 (73.33)	
TPOAb antibody (IU/mL)	341.56±28.96	341.91±35.47	<0.001
Tumor diameter (cm)			0.010
<1	58 (68.24)	5 (33.33)	
≥1	27 (31.76)	10 (66.67)	
Multifocality			0.012
Yes	23 (27.06)	9 (60.00)	
No	62 (72.94)	6 (40.00)	
Tumor location			<0.001
Upper/middle portion	77 (90.59)	5 (33.33)	
Lower portion	8 (9.41)	10 (66.67)	
Capsular invasion			0.025
Yes	13 (15.29)	9 (60.00)	
No	72 (84.71)	6 (40.00)	
Calcification			0.023
Yes	41 (48.24)	12 (80.00)	
No	44 (51.76)	3 (20.00)	
Aspect ratio			0.017
<1	56 (65.88)	5 (33.33)	
≥1	29 (34.12)	10 (66.67)	
Morphology			0.035
Regular	42 (49.41)	3 (20.00)	
Irregular	43 (50.59)	12 (80.00)	

Data are reported as means and SD or number (%). Student's *t*-test and chi-squared test.

### Preoperative ultrasound features of the CLNM and non-CLNM groups among patients with PTC and HT

The CLNM and non-CLNM groups differed significantly in tumor diameter, multifocality, location, capsular invasion, calcification, aspect ratio, and morphology (all P<0.05; Table 2).

### Univariate and multivariate logistic regression analyses of CLNM in patients with PTC and HT

Univariate logistic regression analysis of CLNM in patients with PTC and HT showed that age, gender, tumor diameter, TPOAb antibody status, multifocal lesions, tumor location, capsular invasion, calcification, aspect ratio ≥1, irregular morphology, and tumor diameter ≥1 cm were strongly correlated with CLNM in patients with PTC and HT (all P<0.05; Table 3).

**Table 3:** Univariate logistic regression analysis of central lymph node metastasis (CLNM) in patients with papillary thyroid cancer (PTC) combined with Hashimoto's thyroiditis (HT).

Factor	β	SE	Wals	P	OR	95%CI
Age	-0.061	0.029	4.406	0.036	0.941	0.889-0.996
Gender	1.417	0.624	5.150	0.023	4.125	1.213-14.027
Tumor diameter ≥1 cm	1.458	0.595	5.998	0.014	4.296	1.338-13.796
TPOAb antibody	0.040	0.012	12.184	<0.001	1.041	1.018-1.065
Multifocality	1.397	0.581	5.785	0.016	4.043	1.295-12.623
Tumor location subordinate	2.958	0.662	19.971	<0.001	19.250	5.261-70.431
Capsular invasion	1.306	0.607	4.629	0.031	3.692	1.123-12.136
Presence of calcification	1.457	0.681	4.577	0.032	4.293	1.130-16.308
Aspect ratio ≥1	1.351	0.594	5.182	0.023	3.862	1.207-12.362
Irregular morphology	1.363	0.681	4.005	0.045	3.907	1.028-14.842

Multivariate logistic regression analysis of CLNM in patients with PTC and HT showed that capsular invasion, aspect ratio ≥1, calcification, and irregular morphology were independent risk factors for CLNM in patients with PTC and HT (all P<0.05; Table 4).

**Table 4:** Multivariate logistic regression analysis of central lymph node metastasis (CLNM) in patients with papillary thyroid cancer (PTC) combined with Hashimoto's thyroiditis (HT).

Factor	β	SE	Wals	P	OR	95%CI
Capsular invasion	1.950	0.860	5.149	0.023	7.032	1.304-37.913
Presence of calcification	2.365	0.889	7.082	0.008	10.647	1.865-60.785
Aspect ratio ≥1	1.763	0.714	6.091	0.014	5.827	1.437-23.625
Irregular morphology	1.759	0.794	4.913	0.027	5.806	1.226-27.502

### Nomogram construction and validation

**Figure 1:**
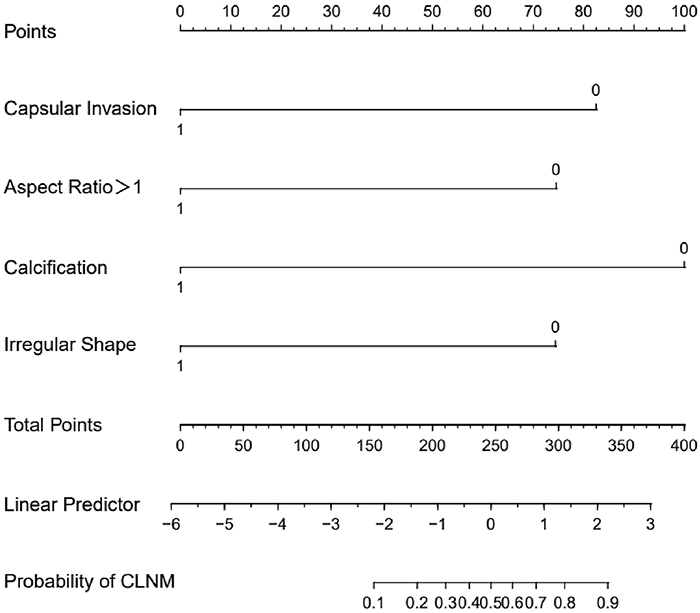
An intuitive nomogram for predicting the occurrence of central lymph node metastasis (CLNM) in patients with papillary thyroid cancer (PTC) combined with Hashimoto's thyroiditis (HT).

Based on the results of multivariate logistic regression analysis, four indicators including capsular invasion, aspect ratio ≥1, calcification, and irregular morphology were used to construct an intuitive nomogram for predicting the occurrence of CLNM in patients with PTC and HT (Figure 1). A single score for each risk factor was obtained based on the nomogram scale corresponding to that factor. The total score indicated the incidence of CLMN in the corresponding patient. The higher the total score, the greater the likelihood of CLMN risk.

## Discussion

PTC metastasis mostly occurs in the central lymph nodes and is accompanied by few distant metastases and reduced mortality. At present, the rate of CLNM detection by ultrasound is unsatisfactory (14). Therefore, it would be valuable to preoperatively identify patients with PTC who are at a greater risk of developing CLNM. In recent years, prediction models and risk factor analyses for diverse diseases based on clinical data have been increasingly developed. As reported, the CLNM predictive model constructed and visualized according to the evaluated risk factors is a functional and convenient tool for predicting CLNM in PTC cases clinically (15). The preoperative nomogram presented good calibration and discrimination for both the training and validation cohorts along with the combined dataset (16). In this study, we aimed to explore the construction and application of a nomogram model for early-stage CLNM in patients with PTC and HT.

A lower proportion of patients with PTC and HT were females. In addition, those with PTC and HT had an elevated tumor aspect ratio, a reduced CLNM rate, a smaller tumor diameter, a lower probability of capsular invasion, and elevated serum TgAb and TPOAb levels. Patients with PTC and HT exhibited less aggressive characteristics and more favorable outcomes compared with those without HT, suggesting that autoimmune thyroiditis protects against thyroid cancer (17). Kim et al. (18) reported similar results in their study, in which the coexistence of HT with PTC was negatively associated with CLNM after adjusting for gender, age, tumor size, and multifocality through a multivariate logistic analysis. Similarly, Jara et al. (19) reported that patients with HT have a smaller tumor size and reduced incidence of angioinvasion, extrathyroidal extension, and CLNM. Additionally, one study identified that the number of lymph nodes during central lymph node dissection was elevated in PTC patients with HT, although it did not enhance the positive lymph node detection rate (20).

We also found significant differences between the CLNM and non-CLNM groups in terms of age, gender, TPOAb status, tumor diameter, multiplicity, tumor location, capsular invasion, calcification, aspect ratio, and morphology, suggesting that these factors may influence the occurrence of lymph node metastasis. In the univariate logistic regression analysis, age, gender, tumor diameter, TPOAb antibody status, multifocal lesions, tumor location, capsular invasion, calcification, aspect ratio ≥1, irregular morphology, and tumor diameter ≥1 cm were strongly correlated with CLNM in patients with PTC and HT. In the multivariate logistic regression analysis, capsular invasion, aspect ratio ≥1, calcification, and irregular morphology were identified as independent risk factors of CLNM in patients with PTC and HT. A nomogram was constructed to visualize and graphically calculate the probability of a patient with PTC and HT developing CLNM.

Several previous studies reported similar results to our findings. For instance, based on univariate and multivariate logistic regression analyses, Zhao et al. (16) found that a normal body mass index, younger age, aspect ratio >1, larger maximum diameter, BRAF V600E mutation, left lobe tumor, capsular invasion, and calcification were risk factors for CLNM in patients with PTC and HT. Based on the findings of binary logistic regression analysis, Chen et al. (21) reported that tumor size >10 mm and CEUS hypoenhancement are independent characteristics of CLNM in patients with PTC and HT. Another study demonstrated that lower tumor location, irregular margins, very high serum TgAb levels, and CLNM microcalcification are linked with CLNM in patients with PTC and HT. The constructed nomogram maintained satisfactory discriminative power in predicting the CLNM risk before surgery in these patients (22).

To conclude, this study confirmed that capsular invasion, aspect ratio ≥1, calcification, and irregular morphology are independent risk factors for CLNM development in patients with PTC and HT. The nomogram model developed in this study has great potential for clinical applications. Our findings provide crucial evidence for constructing a nomogram model. In clinical practice, the nomogram model can assist clinicians in accurately assessing the risk of lymph node metastasis in patients with PTC and HT, thereby assisting with formulating individualized treatment strategies, avoiding over- or under-treatment, and improving patients' treatment outcomes and quality of life.

One key limitation of this study was our small sample size. We also mainly focused on patients with PTC complicated by HT and did not fully explore the risk factors in patients with PTC only. Future studies should ensure a large sample size and apply a multicenter study design to further verify and optimize the nomogram model to improve its predictive accuracy. Additionally, studies involving patients with PTC only should be conducted to determine the similarities and differences in risk factors for CLNM in these patients compared to patients with PTC and HT.

## Data Availability

All data generated or analyzed during this study are included in this published article.
